# Mesocarnivores and macroparasites: altitude and land use predict the ticks occurring on red foxes (*Vulpes vulpes*)

**DOI:** 10.1186/s13071-017-2113-9

**Published:** 2017-04-05

**Authors:** Attila D. Sándor, Gianluca D’Amico, Călin M. Gherman, Mirabela O. Dumitrache, Cristian Domșa, Andrei Daniel Mihalca

**Affiliations:** Department of Parasitology and Parasitic Diseases, Faculty of Veterinary Medicine, University of Agricultural Sciences and Veterinary Medicine, Calea Mănăştur 3-5, Cluj Napoca, Romania

**Keywords:** Red fox, Tick-infestation, *Vulpes vulpes*, Land use, Climate, Altitude

## Abstract

**Background:**

The red fox *Vulpes vulpes* is the most common mesocarnivore in Europe and with a wide geographical distribution and a high density in most terrestrial habitats of the continent. It is fast urbanising species, which can harbor high numbers of different tick species, depending on the region. Here we present the results of a large-scale study, trying to disentangle the intricate relationship between environmental factors and the species composition of ectoparasites in red foxes. The samples were collected in Transylvania (Romania), a region with a diverse geography and high biodiversity. The dead foxes (collected primarily through the National Surveillance Rabies Program) were examined carefully for the presence of ticks.

**Results:**

Ticks (*n* = 4578) were found on 158 foxes (out of 293 examined; 53.9%). Four species were identified: *Dermacentor marginatus*, *Ixodes canisuga*, *I. hexagonus* and *I. ricinus*. The most common tick species was *I. hexagonus* (mean prevalence 37.5%, mean intensity 32.2), followed by *I. ricinus* (15.0%; 4.86), *I. canisuga* (4.8%; 7.71) and *D. marginatus* (3.7%; 3.45). Co-occurrence of two or more tick species on the same host was relatively common (12.6%), the most common co-occurrence being *I. hexagonus - I. ricinus*. For *D. marginatus* and *I. canisuga* the highest prevalence was recorded in lowlands, for *I. hexagonus* in hilly areas, while for *I. ricinus* in mountains.

**Conclusions:**

Altitude influenced the intensity of parasitism, with highest intensity observed for all *Ixodes* species in hilly areas. *Dermacentor marginatus* occurred only in lowlands, *I. canisuga* in lowlands and hilly areas while the other two species occurred in all of the regions studied. Foxes from lower altitudes had the most tick species associated, with most incidences of co-parasitism also recorded here. Land use affected tick-species composition, with the presence of *D. marginatus* strongly associated with the extension of arable areas and lack of forests. The presence of *I. hexagonus* was determined only by the extent of arable lands. As foxes are frontrunners of wildlife urbanization process, with a continuous increase of their numbers in urban areas, the knowledge of their ticks’ ecology (and the pathogens vectored by these) is of utmost importance.

## Background

Ticks are probably responsible for transmitting the highest number of pathogens, being competent vectors for a large number of piroplasms, viruses or bacteria. They are the medically most important group of arthropods in Europe, with an estimated 85,000 cases reported only for Lyme borreliosis [1], having also high associated health-care costs for most tick-borne diseases. In addition, ticks transmit a wide range of pathogens affecting livestock and companion animals [2, 3].

The medical importance of tick-borne diseases is acknowledged and most vectored diseases have a long epidemiological history; still, several tick-borne diseases show an emerging pattern all over Europe (and some even globally). Tick and host distributions are generally known in Europe [1] and the most important hosts of the different tick species have been identified for long time [4]; however, the delicate relationship that governs this co-occurrence still requires further studies.

The red fox (*Vulpes vulpes*) is the most common mesocarnivore in Europe, with a wide geographical distribution and a high density in most terrestrial habitats of the continent [5–8]. Being a species which successfully adapted to most habitats (even the most highly altered ones, like cities), it is a prime candidate for harboring and distributing en-mass different parasite species [9–11] in areas where high density human population and/or domesticated livestock occur [12–15]. It is a sedentary and territorial species, with a relatively small home range [12, 16]. Thus, as host for ticks (and pathogens transmitted by ticks) the red fox has an utmost importance, not only from biological, but also from epidemiological perspective [13, 17]. Moreover, foxes can harbor high numbers of different tick species and the faunal composition of these tick loads may differ from site to site [18–20]. While ticks may not pose significant health risk directly related to hematophagy, their importance resides in their capacity of transmitting pathogens. Tick communities harbored by foxes were extensively studied in western [19–24] and central Europe [17, 18, 25–27]. However, studies are scant in the eastern part of the continent. There is no large-scale study published for Romania on ticks of foxes, with the only paper on the subject being a list of records [28]. The red fox is the most common wild carnivore in the country, occurring in all terrestrial habitats [29]. Here we present the results of a study targeting the fox-tick relationship in the landscape of the north-western and central part of Romania (Transylvania), an area which presents a high diversity of landscape features (mostly covered by the Carpathians, but incorporating parts of the Pannonian Plain, with altitudes ranging from 90 to 2000 m above sea level, a.s.l.), providing an excellent background to study the influence the environment may have on the composition and tick-burdens in red foxes. Our study is the first of this kind which tries to disentangle the intricate relationship between environmental factors and the species’ composition of ectoparasites in a common mesocarnivore. Also we highlight the importance of the altitude and land use for this host and the parasite species harboured in the context of vector-borne disease potential they may pose.

## Methods

### Study region

**Fig. 1:**
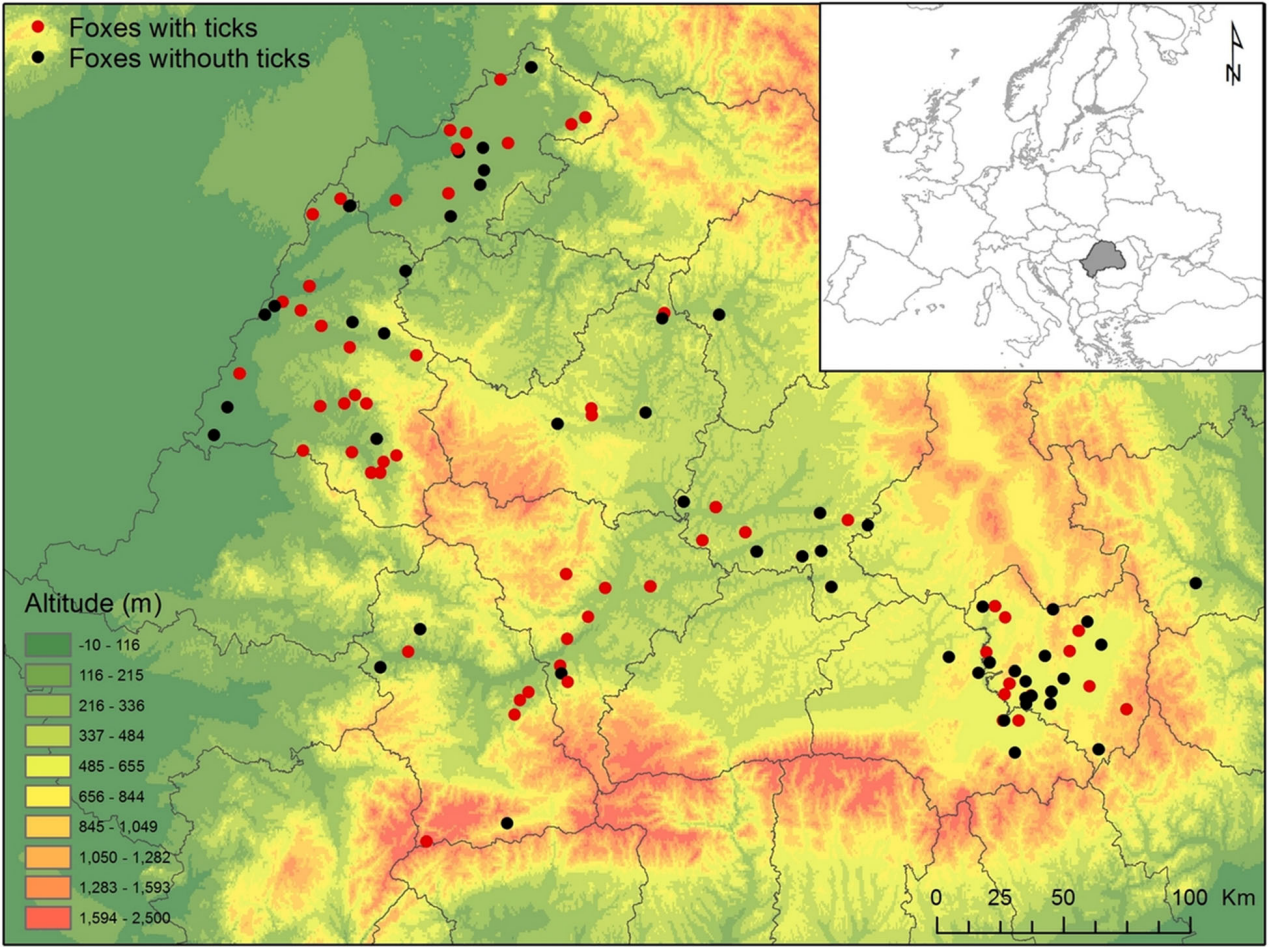
Geographical distribution of ticks collected from red foxes (*Vulpes vulpes*) in Transylvania, Romania

The samples were collected in the historical region of Transylvania, in Romania (Fig. 1). The region has a diverse geography (and implicitly climate), dominated by the Carpathian Mountains (Transylvanian Alps). It is one of the wildest areas in Europe, with a high degree of natural and semi-natural vegetation and an exceptional biodiversity [30]. The area has high forest cover (42%), with most regions still maintaining traditional low intensity or subsistence agriculture, favoring a mosaic-like landscape composition [31, 32]. The sample collection sites are distributed in most important land use types, and cover all altitude ranges (91–1789 m a.s.l.) where red foxes may occur in the region. The samples were collected from red foxes, received as corpses from the National Surveillance of Rabies Program managed by the Romanian Agency for Veterinary and Public Health, based on sub-samples of animals resulted from commercial hunting and pest-control activities. All the animals received were collected using guns by licensed hunters (professional and amateur alike) in the course of organized game-management activities. Corpses of animals free of rabies were transported to our laboratory according to the current laws on animal corpse transport and zoonotic risks. A smaller percent of the controlled animals was received as road-kills (*n* = 12; 4.1%) (the University of Agricultural Sciences and Veterinary Medicine holds a national-wide license and is fully equipped for collection and disposal of biohazardous and medical waste, including animal carcasses). No live fox was harmed for the sake of this study.

### The tick data

The foxes were stored in individual plastic bags deep frozen until examination. The collection period lasted 24 months, between May 2010 and April 2012. The whole body surface of each fox was examined carefully for the presence of ticks. The parasites were stored in 87% ethanol in separate vials from each host. While special care was taken to collect all ticks, we are not fully confident that all individuals of larval stages were found in each animal. Ticks were identified to species and development stage using morphological keys [33, 34].

### Land use

The location of each collection site was geo-referenced and we collected environmental predictors using a grid 2 × 2 km cells containing the geo-referenced coordinates of the collection site. These cells had 400 ha, similar to the average red fox home range from semi-natural and natural habitats from all over Europe (mean 413.42 ha; range 12.95–1990.00 ha; standard deviation, SD, 393.1192 ha, *n* = 84 studies, [5, 6, 8, 35]). These cells were the unit for parasitological (mean intensity, frequency, prevalence of ticks on foxes) and land use data. To assess land use, we used CORINE LandCover (European Environment Agency, http://www.eea.europa.eu/). We used five predictors (altitude, per cent of arable land/grassland/urbanized areas/forest cover; see Table 1 for the associated CORINE LandCover categories). There was no statistical difference (*χ* ^2^ = 0.2258, *df* = 3, *P* < 0.97) between land use composition inside the sampled 2 × 2 km plots and the overall land use composition of the region, thus we consider that our results may be generalized for the whole region.

**Table 1:** Correspondence between CORINE LandCover categories and land use types used in this study

Code Level 3	Label level 3	Assigned land use type
111	Continuous urban fabric	Urban
112	Discontinuous urban fabric	Urban
121	Industrial or commercial units	Urban
122	Road and rail networks and associated land	Urban
123	Port areas	Urban
124	Airports	Urban
131	Mineral extraction sites	Urban
132	Dump sites	Urban
133	Construction sites	Urban
141	Green urban areas	Urban
142	Sport and leisure facilities	Urban
211	Non-irrigated arable land	Arable
212	Permanently irrigated land	Arable
213	Rice fields	Arable
221	Vineyards	Arable
222	Fruit trees and berry plantations	Arable
223	Olive groves	Arable
231	Pastures	Grassland
241	Annual crops associated with permanent crops	Arable
242	Complex cultivation patterns	Arable
243	Land principally occupied by agriculture, with significant areas of natural vegetation	Grassland
244	Agro-forestry areas	Forest
311	Broad-leaved forest	Forest
312	Coniferous forest	Forest
313	Mixed forest	Forest
321	Natural grasslands	Grassland
322	Moors and heathland	Grassland
323	Sclerophyllous vegetation	Grassland
324	Transitional woodland-shrub	Forest
333	Sparsely vegetated areas	Grassland

### Statistical procedures

Mean intensity, frequency, prevalence and its 95% confidence interval (CI) were calculated using the software Quantitative Parasitology 3.0 [36]. Sample prevalence data were analyzed using Fisher’s exact test. Relationship between tick prevalence and environmental predictors (land use and altitude) was tested using Spearman’s rank correlation. Differences were considered significant when *P* < 0.05.

## Results

### Tick parasitism

**Fig. 2:**
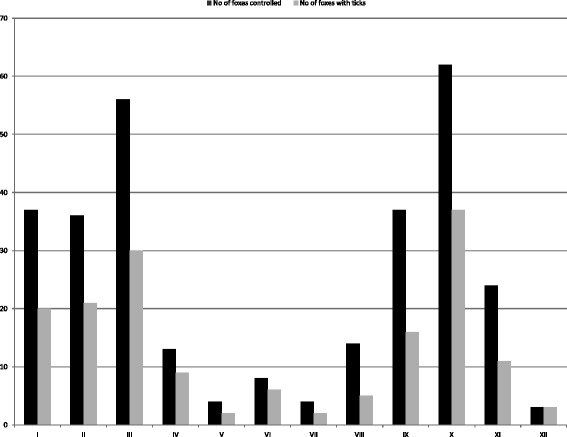
Monthly distribution of foxes analyzed and foxes with ticks

**Fig. 3:**
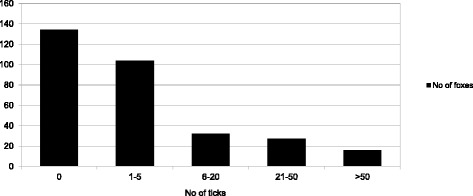
Distribution of infestation-level frequency of ticks found on foxes

Altogether 293 foxes collected from 10 counties and at least 186 different locations were examined for ticks (Fig. 1). For a number of 48 foxes (16.4%), the exact collection site was unknown, thus these were excluded from the geographical analysis. Foxes were collected in each month; however, their distribution was not even, with most foxes hunted in autumn and winter months (due to hunting regulations, Fig. 2). Ticks (*n* = 4578) were found on 158 foxes (53.9%), with four tick species identified: *Dermacentor marginatus*, *Ixodes canisuga*, *I. hexagonus* and *I. ricinus* (Table 2). The most common tick species was *I. hexagonus* (mean prevalence 37.5%, CI: 31.4–43.5; mean intensity 32.2), followed by *I. ricinus* (mean prevalence 15.0%, CI: 10.1–19.8; mean intensity 4.86), *I. canisuga* (mean prevalence 4.8%, CI: 2.1–7.5; mean intensity 7.71), and *D. marginatus* (mean prevalence 3.7%, CI: 1.3–6.1, mean intensity 3.45). Most foxes (*n* = 104, 65.8%) had a low intensity of parasitism, with less than 5 ticks, while 7 foxes hosted more than 100 ticks, with one individual holding 2229 ticks, all larvae (Fig. 3). Co-occurrence of two or more tick species on the same host was relatively common, with 12.6% of foxes with ticks harboring more than one species (Table 3). The most common co-occurrence of different tick species on single foxes was *I. hexagonus - I. ricinus* (40% of all co-parasitism cases).

**Table 2:** The tick infestations of red foxes (*Vulpes vulpes*) in Transylvania, Romania

Tick species	Number of foxes with ticks	Male	Female	Nymphs	Larvae	Total
*Ixodes canisuga*	14	–	26	66	17	109
*Ixodes hexagonus*	110	–	12	144	1,827^a^	1,983^a^
*Ixodes ricinus*	44	62	146	9	2	219
*Dermacentor marginatus*	11	20	18	–	–	38
Total	158^b^	82	202	219	1846	2349

^a^Does not include the 2229 larvae of *I. hexagonus* collected from one individual fox

^b^Including foxes harboring more than one tick species

**Table 3:** Mixed infestations of ticks found on red foxes

Tick species present	No. of foxes	Percentage^a^
One tick species	139	87.3
*I. hexagonus - I. ricinus*	8	5.0
*I. hexagonus - D. marginatus*	6	3.8
*I. ricinus - I. canisuga*	3	1.9
*I. ricinus - D. marginatus*	1	0.6
*I. hexagonus - I. ricinus - D. marginatus*	1	0.6
Foxes without ticks	135	46.1

^a^Only foxes with ticks considered

### Importance of altitude and land use

Highest prevalence of tick occurrence was noted in foxes collected on lowlands (altitude below 200 m), followed by foxes in hilly areas (200–700 m) (mostly *I. hexagonus*). Lowest number of ticks was found in foxes of mountainous regions (above an altitude of 700 m). For two species (*D. marginatus* and *I. canisuga*) the highest prevalence was recorded in lowlands, for *I. hexagonus* in hilly areas, while for *I. ricinus* in mountain areas. However, none of these differences were significant.

**Fig. 4:**
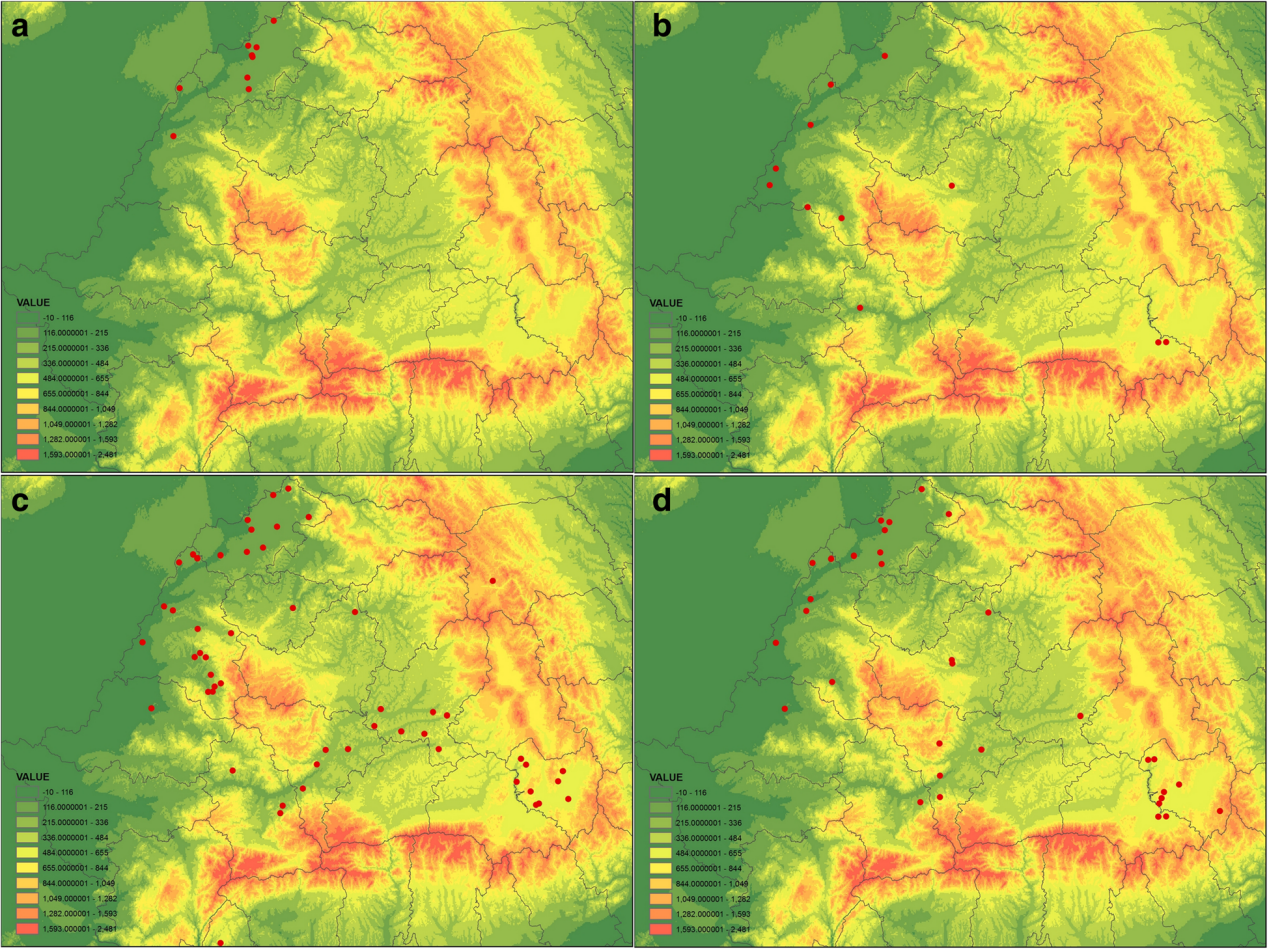
Geographical distribution of tick species infesting red foxes. **a** *Dermacentor marginatus*. **b** *Ixodes canisuga*. **c** *I. hexagonus.* **d** *I. ricinus*

Altitude also influenced the intensity of parasitism, with highest intensity observed for all but one tick species (*D. marginatus*, found only in lowlands) in hilly areas (Table 4). The geographical distribution of different tick species was also determined by altitude, with *D. marginatus* occurring only in lowlands (mean altitude 128.9 m, CI: 116–130, *n* = 11); *I. canisuga* in lowlands and hilly areas (mean altitude 266.2 m, CI: 112.2–492.7, *n* = 14; Fig. 4). The other two species occurred in all the regions studied, but with a different mean altitude of occurrence (mean altitude for *I. hexagonus* 322.2 m, CI: 156.2–380.5, *n* = 84; for *I. ricinus* 404.3 m, CI: 148.7–522.5, *n* = 42; Fig. 4). For all but one species (*I. hexagonus*) altitude was the most important factor determining presence (Table 5). Foxes from lower altitudes had the most tick species associated; with most incidences of co-parasitism noted below the elevation of 300 m (mean altitude for co-parasitism incidences 261.5 m, CI: 118.2–387.7, *n* = 18).

**Table 4:** Mean prevalence (Prev, %) and mean intensity (Int, *n*) of tick species found on foxes according to the altitudinal regions

Altitude region	*D. marginatus*		*I. canisuga*		*I. hexagonus*		*I. ricinus*	
	Prev	Int	Prev	Int	Prev	Int	Prev	Int
Lowlands	14.5	3.4	9.2	6.1	36.0	13.8	17.1	1.7
Hilly	–	–	4.6	9.4	72.0	29.3	16.1	7.8
Mountain	–	–	–	–	15.8	10.3	27.8	1.6

**Table 5:** Relationship between tick species prevalence and environmental predictors

Tick species	Altitude	Land use			
		Arable	Urban	Forest	Grassland
*I. canisuga*	-0.09	0.09	0.04	-0.13*	-0.02
*I. hexagonus*	-0.1	0.12*	0.05	-0.1	-0.05
*I. ricinus*	0.08	-0.05	-0.04	0.1	-0.08
*D. marginatus*	-0.19*	0.15*	0	-0.13*	-0.03
Mixed infestations	-0.17*	0.23*	-0.11	-0.12*	-0.14*

*Correlations indicated in bold are significant at *P* < 0.05

Land use also influenced tick-species composition, with occurrence of *D. marginatus* highly associated to the extension of arable areas and lack of forests inside the 2 × 2 km cell, while the presence of *I. hexagonus* was determined only by the extent of arable lands. Presence of forests was correlated with the lack of *D. marginatus* and *I. canisuga* (Table 5). We found no influence of the extent of urban areas inside the sample cell on neither of the identified tick species. No effect of seasonality on the geographical distribution was observed.

## Discussion

In this study ticks collected from 293 red foxes from 183 individual locations were analyzed. We found that foxes had diverse tick assemblages according to season, land use and altitude. More than half of the investigated foxes were hosting ticks. Similar prevalence values were reported from other large-scale studies [18, 22, 26, 36, 37].

Red foxes are important hosts for ticks all over Europe, with at least 17 tick species known to occur on foxes, with regional differences among tick faunas. There are three species of ticks (*I. canisuga*, *I. hexagonus* and *I. ricinus*) which are commonly found on foxes in most studied regions, but their prevalence and abundance may exhibit large variations [26, 36, 37]. In addition to these common species, in most regions there are several other tick species which may be the locally dominant fox ticks. Foxes from the Western Mediterranean region are parasitized primarily by *Rhipicephalus* species (*R. pusillus*, *R. sanguineus* (*s.l*.) and *R. turanicus*) and *I. ventalloi* [19, 22, 36–39]. Further north, in regions with climate determined by the Atlantic Ocean, the tick fauna of foxes includes almost exclusively *I. canisuga*, *I. hexagonus* and *I. ricinus* [21, 40]. In central Europe, *Haemaphysalis concinna* and *D. reticulatus* appear complementary to these three [18, 25, 26, 41, 42]. In southern Europe (Italy, Croatia, Turkey) most foxes are hosting (in addition to the three main species and the typical Mediterranean *Rhipicephalus* spp.) also *Haemaphysalis* species (*H. erinacei*, *H. inermis*, *H. parva*, *H. punctata* and *H. sulcata*) and rarely, *D. marginatus* [27, 43–45].

The tick fauna of red foxes from Transylvania is similar regarding species composition to most central and western European studies, as the three most commonly found tick species are *I. hexagonus*, *I. canisuga* and *I. ricinus.* However, the fourth species (*D. marginatus*) was reported only from the southern part of the continent [44]. While in most studies *I. ricinus* or *I. canisuga* are the dominant tick species in foxes, in our study *I. hexagonus* was found to have the highest prevalence and intensity. Similar prevalences of *I. hexagonus* were reported only by Harris & Thompson [21] for suburban foxes in London and by Dominguez [36] for the mountainous region of Burgos in Spain, however in both cases one of the other two *Ixodes* species was the dominant tick. *Ixodes hexagonus* is a burrow-dwelling tick, adapted to parasitize mammals typically using underground burrows (carnivores and hedgehogs, *Erinaceus* spp.). While Sobrino et al. [19] suggested that the geographical distribution of *I. hexagonus* is not strictly limited by climate (because of the buffering effect of the microclimate of the host’s burrows) there are major differences among prevalences on the continent. While most studies failed to confirm the presence of *I. hexagonus* in southern Europe and it was scarcely found in central Europe, it was reported in high numbers from the mountainous areas of Northern Spain [36] and in countries with moist Atlantic climate (UK) or northern Germany [21, 26]. Although all over in Europe foxes use underground burrows for breeding, it seems that this tick species prefers areas with higher atmospheric humidity. The high prevalence of *I. hexagonus* in central Romania is probably linked to higher atmospheric moisture levels compared to neighboring countries, due to higher elevations (and associated higher levels of rainfall) of fox occurrences than in Croatia, Hungary or Germany.

*Ixodes canisuga* is another typical burrow-tick, commonly occurring in foxes [21]. The prevalence recorded in our study is similar to the ones found in Italy, UK or Spain, but lower than in Hungary [18] or Germany [26]. The prevalence of *I. ricinus* on foxes in our study was lower than in those studied in Germany [26] and Hungary [18], but was higher than in most other studies reporting this species from foxes [25, 37, 39]. Most likely, this is determined by the high frequency of forest cover in our study area, as this tick species shows high affinity towards forests [46]. *Dermacentor marginatus* is a tick with an eastern European distribution, and thus rarely recorded on foxes, with only one published study reporting a prevalence similar to the one recorded in Romania [44].

The red fox is the most common carnivore of Europe, and they are commonly encountered in most habitats and elevations from seashores to alpine regions, with winter harshness being the only known limit for its occurrence [35]. The species is the most common carnivore in Romania, and its distribution covers the territory of the entire country [29]. We found ticks on foxes collected in an altitudinal range from 91 to 1789 m a.s.l. (covering lowlands to high alpine regions in Romania) and the parasite burdens were diverse, seemingly being influenced primarily by altitude. Foxes from lowlands had the highest prevalence of tick parasitism and diversity, but a low intensity, which was the highest in hilly areas. While the higher overall prevalence may be caused by the overall higher number of tick species present at low altitudes, we have no explanation for the lower intensity. There were significant differences in tick species’ composition in red foxes in relation to altitude, land use and habitat composition. One of the tick species encountered (*D. marginatus*) occurred only at low altitudes (<200 m), while another (*I. canisuga*) also was limited by altitude, with an upper limit of occurrence at 521 m a.s.l. Although the other two tick species showed a higher tolerance towards altitude, their median occurrences showed that their distributional optima were different. *Ixodes hexagonus* was found most commonly in hilly areas (75% of all occurrences within the range of 211–509 m a.s.l.), with the highest prevalence and intensity found also in this range. *Ixodes ricinus* had a similar wide distributional range, however, its highest prevalence was found in mountain areas (Table 4).

Tick distribution showed a regional trend, with different habitats and land use generally linked to different tick faunal compositions. High percentage of forest cover inside the sample cell was negatively correlated with the presence of *D. marginatus* and *I. canisuga*, with *D. marginatus* common in habitats containing high percentage of arable land (Table 5). Although *D. marginatus* is primarily a tick species of grasslands and open landscapes [47], hence its wide association with the presence of agricultural areas is easily understood, we have no such explanation for *I. canisuga*. Arable areas also favored the presence of *I. hexagonus*, this being the only land use category predicting its occurrence. As most tick species were positively correlated with the presence of arable lands, this is the most important predictor for the high incidence of mixed infestations as well, with all the other predictors negatively influencing the occurrence of multiple infestations in foxes. No visible influence of any predictor was established for *I. ricinus*. This denotes the wide generalist character of this species, being by far the most common tick in Romania [46].

The four tick species found in Transylvania are common parasites of mostly wild and domestic carnivores [48]. Three of these, *D. marginatus*, *I. hexagonus* and *I. ricinus*, are commonly occurring on small mammals and humans [49, 50], while *D. marginatus* and *I. ricinus* also occur on ruminants [51]. Their epidemiologic importance is long established, with these species being vectors of certain *Anaplasma* spp. [52, 53], *Babesia* spp., *Rickettsia* spp., zoonotic viruses and - except for *D. marginatus - Borrelia* spp. [1]. Their geographical distribution range has been also established for a long time, but their ecology and the influence of bio-climatic factors on their distribution and population dynamics still lack details. By elucidating the importance of altitude and habitat structure in shaping the tick fauna of red foxes we provide new tools for establishing the epidemiological importance of this carnivore host. As foxes are frontrunners of the wildlife urbanization process [12], with a continuous increase of their numbers in urban areas [13], the knowledge of their ticks’ ecology (and the pathogens vectored by these) is of utmost importance.

## Conclusions

Altitude influenced the intensity of parasitism, with highest intensity observed for all *Ixodes* species in hilly areas. *Dermacentor marginatus* occurred only in lowlands, *I. canisuga* in lowlands and hilly areas while the other two species occurred in all the regions studied. Foxes from lower altitudes had the most tick species associated, with most incidences of co-parasitism noted at low elevations. Land use affected tick species composition, with the presence of *D. marginatus* strongly associated with the extension of arable areas and lack of forests. The presence of *I. hexagonus* was determined only by the extent of arable lands.
